# Getting to the heart of the matter: Does aberrant interoceptive processing contribute towards emotional eating?

**DOI:** 10.1371/journal.pone.0186312

**Published:** 2017-10-18

**Authors:** Hayley A. Young, Claire Williams, Aimee E. Pink, Gary Freegard, Amy Owens, David Benton

**Affiliations:** Department of Psychology, Swansea University, Swansea, United Kingdom; Medical University of Vienna, AUSTRIA

## Abstract

According to estimates from Public Health England, by 2034 70% of adults are expected to be overweight or obese, therefore understanding the underpinning aetiology is a priority. Eating in response to negative affect contributes towards obesity, however, little is known about the underlying mechanisms. Evidence that visceral afferent signals contribute towards the experience of emotion is accumulating rapidly, with the emergence of new influential models of ‘active inference’. No longer viewed as a ‘bottom up’ process, new interoceptive facets based on ‘top down’ predictions have been proposed, although at present it is unclear which aspects of interoception contribute to aberrant eating behaviour and obesity. Study one examined the link between eating behaviour, body mass index and the novel interoceptive indices; interoceptive metacognitive awareness (IAw) and interoceptive prediction error (IPE), as well as the traditional measures; interoceptive accuracy (IAc) and interoceptive sensibility (IS). The dissociation between these interoceptive indices was confirmed. Emotional eaters were characterised by a heightened interoceptive signal but reduced meta-cognitive awareness of their interoceptive abilities. In addition, emotional eating correlated with IPE; effects that could not be accounted for by differences in anxiety and depression. Study two confirmed the positive association between interoceptive accuracy and emotional eating using a novel unbiased heartbeat discrimination task based on the method of constant stimuli. Results reveal new and important mechanistic insights into the processes that may underlie problematic affect regulation in overweight populations.

## Introduction

Although deficits in emotion regulation are a commonly used explanation for the development and maintenance of obesity [1], the exact process by which emotions affect eating behaviour remains an unanswered question. Over the past decade our understanding of the neurobiology of both obesity and emotion has increased dramatically. The recognition that visceral afferent signals are pertinent to the emotional experience (a process referred to as interoception: the perception and interpretation of bodily signals) has shed new light on the mechanisms surrounding emotional disorders [2–6] and has the potential to enhance our understanding of obesity. The self–evaluative element of interoception [operationalized using the Interoceptive Awareness subscale of the Eating Disorders Inventory (EDI)] is altered in a range of eating pathologies including obesity [7]. However, interoception is no longer considered a unitary construct and it is unclear which aspects of interoception contribute to aberrant eating behaviour and obesity: thus a more complete understanding of the inter-relations between interoception, obesity and emotion is required.

A limited literature has examined the relationship between interoception and eating behaviour but the majority of studies have used self-evaluation measures [7]. The few studies that have considered objective measurements have focused exclusively on ‘bottom up’ signalling and have found mixed results. For example, in an undergraduate sample, a higher interoceptive accuracy, operationalized as one’s ability to detect their heartbeat, was associated with a tendency to eat for physical rather than emotional reasons [8] and with a lower body weight [9]. Conversely, patients with bulimia had normal heartbeat detection (HBD) accuracy but reduced self–evaluated interoceptive sensibility according to the Eating disorders inventory (EDI) [10], whereas patients with anorexia had both reduced HBD and EDI [11]. Women who had recovered from bulimia nervosa had reduced HBD compared to healthy controls [12], as did those with anorexia nervosa [13]. Other studies involving clinical populations have found no difference between eating disordered individuals and healthy controls [14, 15]. There are a number of possible explanations for these inconsistencies. Firstly, eating disorder patients often experience comorbid mood and anxiety disorders [16], which are themselves associated with altered heart beat perception [17]. Therefore, the first aim of the present studies was to determine the link between interoception and eating behaviour whilst accounting for differences in anxiety and depression.

A further explanation for these discrepancies is that interoception should not be considered simply as a ‘bottom up’ gathering of evidence. As new models of ‘active inference’ begin to take shape, it is increasingly clear that ‘top down’ interoceptive inferences play a pivotal role. Within these models interoception is conceptualised as a inferential process [5], whereby emotions arise as a result of inferences about the causes of interoceptive afferents [6]. ‘Top down’ inference is constrained according to prior likelihood: hypotheses are generated based on prior experience of the cause of interoceptive signals. Predictions are then tested against actual incoming afferent information with any ‘mismatch’ giving rise to ‘prediction error’. The eventual perceptual experience will be the hypothesis with the highest posterior probability (the probability that the hypothesis is correct after the incoming interoceptive evidence has been considered). The extent to which ‘bottom up’ interoceptive signals, relative to ‘top down’ prior beliefs, influence emotion depends upon the certainty or ‘precision’ awarded to them [18]. Importantly, precision also has a metacognitive component, such that prior beliefs about precision allocation might also influence emotional content [19].

Within this framework unwanted / unneeded emotional experiences arise as a result of aberrant interoceptive predictions [20]. Understanding how these processes contribute towards eating behaviour has the potential to redefine current thinking about the role of interoceptive signalling. Therefore the present studies determined the relative contribution of the following interoceptive indices to aberrant eating behaviour (the propensity to eat in response to emotional cues–emotional eating (EE), external stimuli (EX)) and being overweight: (1) *interoceptive accuracy* (objective heartbeat detection performance), (2) *interoceptive awareness* (meta-cognitive awareness of interoceptive accuracy, confidence-accuracy correspondence), (3) *interoceptive sensibility* (self-evaluated interoceptive *belief*, gauged using interviews/questionnaires) and (4) *interoceptive prediction error* (the difference between objective interoceptive accuracy and subjective interoceptive sensibility) [21].

As traditional heartbeat tracking and heartbeat detection tasks have recently been criticised for producing false-positive and false negative bias respectively [22], we also confirmed our findings in a second sample using a novel heartbeat discrimination task based on the method of constant stimuli. For the first time it is reported that although emotional eaters have a stronger interoceptive signal (confirmed across both interoception tasks), they are characterised by a reduced metacognitive and belief driven awareness of their interoceptive abilities.

## Study 1

### Methods

#### Participants

Thirty six females between 18 and 28 years of age participated in this study (Table 1). The sample size was based on previous research that has considered the association between IAc and eating behaviour [10] and the association between IAw and IPE and emotional behaviour [21]. Participants were excluded if they had a metabolic or cardiovascular disorder, gastrointestinal problems, were pregnant, had a current diagnosis of a mood or eating disorder, and/or were taking medications or herbal supplements to manage body weight or control appetite. BMI ranged from 19.2 to 37.1 (average 23.7) kg/m^2^; 69% of the sample had a normal BMI between 18.5 and 24.9, 20% of the sample were overweight with a BMI between 25 and 30 and the remaining 11% of the sample were obese with a BMI > 30. Participants were instructed to refrain from drinking alcohol and taking part in any physical activity within twenty four hours of the study and to abstain from consuming any food and drink for at least two hours before attending the laboratory.

**Table 1:** Descriptive characteristics of the sample for study 1.

Characteristic	Mean (SD)
**N**	36 Females
**Age (years)**	21.87(1.76)
**Body mass index (kg/m^2^)**	23.70 (4.28)
**Anxiety (VAS)**	30.13 (23.12)
**Depression (VAS)**	37.61 (17.62)
**Confidence (VAS)**	66.50 (19.72)
**Heart rate (BPM)**	68.09 (11.03)
**Emotional eating (EE—DEBQ)**	2.79 (1.05)
**External eating (EX—DEBQ)**	3.27 (0.78)
**Restrained eating (RE—DEBQ)**	3.04 (0.83)
**Interoceptive accuracy (IAc)**	0.63 (0.19)
**Interoceptive awareness (IAw)**	-0.20 (0.48)
**Interoceptive sensibility (IS)**	84.77(18.46)
**Interoceptive prediction error (IPE)**	0.00 (1.24)
**Confidence in HBD performance**	49.29 (21.39)

VAS–Visual analogue scale, BPM–Beats per minute, DEBQ–Dutch eating behaviour questionnaire, HBD–Heart beat detection.

#### Procedure

After providing their written informed consent, participants rated their current mood, and were fitted with a RS800 Polar heart rate monitor electrode transmitter belt (T61) using conductive gel as recommended by the manufacturer. Interbeat interval measurements were collected using the Polar RS800 HR monitor set to R-R interval mode (Polar Electro, Kempele, Finland) at a sampling rate of 1000 Hz. This instrument has been previously validated for the accurate measurement of R-R intervals [23, 24]. Participants then completed the Heartbeat Detection Task according to the Mental Tracking Method [25] (described below). The participants completed the Dutch Eating Behaviour Questionnaire [26] and had their height and weight measured. The procedure was approved by Swansea University department of Psychology ethics committee and was conducted in accordance with the principles laid down by the declaration of Helsinki 2013.

#### Interoceptive accuracy (IAc)

The heartbeat perception task was performed according to the Mental Tracking Method [25] using intervals of 30, 35, 40, and 45 and 50 seconds that were separated by 30 second resting periods. During each trial R-R intervals were recorded and participants were asked to silently count their heartbeats without the use of an exteroceptive aid (such as taking one’s pulse). At the end of each period participants verbally reported the number of counted heartbeats. The participants were not informed about the length of the counting phases nor about the quality of their performance. The following transformation: 1 − (*n*beats_real_ − *n*beats_reported_)/((*n*beats_real_ + *n*beats_reported_)/2) was used on a trial by trial basis to calculate heartbeat tracking scores. These scores were then averaged to form a mean heartbeat tracking score (interoceptive accuracy). The interoception score varied between 0 and 1 with a higher score indicating a better accuracy. This heartbeat detection task is a standard measure used to assess the accuracy of the ability to detect interoceptive signals.

#### Interoceptive awareness (IAw)

At the end of each trial the participants immediately rated his/her confidence in their perceived accuracy of response. This confidence judgement was made verbally and the participants were asked to indicate on a scale of 0–100, how accurate they thought they were with, 0 indicating “Not at all confident” and 100 “Completely confident”. The within-participant Pearson correlation, *r*, between confidence and accuracy provided an index of interoceptive awareness [27].

#### Interoceptive sensibility (IS)

The self-evaluative component was measured using the Multidimensional Assessment of Interoceptive Awareness (MAIA) [28]. This 32-item multidimensional instrument assesses eight concepts related to interoception: (1) *Noticing* (awareness of uncomfortable, comfortable, or neutral body sensations), (2) *Not Distracting* (tendency not to use distraction to cope with discomfort), (3) *Not Worrying* scale (tendency not to experience emotional distress with physical discomfort) (4) *Attention Regulation* (ability to sustain and control attention to body sensations) (5) *Emotional Awareness* (ability to attribute specific physical sensations to physiological manifestations of emotions) (6) *Self-Regulation* scale, (ability to regulate distress by attention to body sensations) (7) *Body Listening* scale, (tendency to actively listen to the body for insight) (8) *Trusting* (the experience of one’s body as safe and trustworthy). In the present study Cronbach's alpha’s were 0.65, 0.69, 0.44, 0.86, 0.79, 0.55, 0.87 and 0.87 respectively. Although designed to assess the multidimensional nature of interoceptive sensibility Cronbach's alpha was highest when all items on the questionnaire were summed: 0.89. As such individual scales were summed to provide a general measure of self–evaluative interoceptive sensibility, however, correlations between each individual subscale and eating behaviour can be found in Supplementary information (S1 File).

#### Interoceptive trait prediction error (IPE)

The IPE was operationalized as the difference between objective interoceptive accuracy and subjective interoceptive sensibility. Z scores were calculated for the interoceptive accuracy and sensibility variables, and IPE values were calculated as the difference between interoceptive sensibility and interoceptive accuracy. This measure has previously been shown to differentiate those with autism from healthy controls and has been shown to correlate with emotional sensitivity and anxiety [21], and the development of abnormal skin sensations [29]. As both over-estimation and under-estimation constitute an increased prediction error, IPE was operationalized using absolute values i.e. higher absolute values constitute increased IPE.

#### Dutch Eating Behaviour Questionnaire (DEBQ)

The tendencies towards emotional eating (EE) (13 items), restrained eating (RE) (10 items) and external eating (EX) (10 items) were measured using the English version of the DEBQ [26]. In the present study Cronbach's alpha’s were 0.90 for restrained, 0.92 for emotional and 0.81 for external eating.

#### Body mass index (BMI)

Body mass was measured using an electronic scale (Kern KMS-TM, Kenr and Sohn GmbH, Germany) that, to avoid problems associated with movement, took 50 assessments over a 5 second period and produced an average value. Height was measured using a portable stadiometer.

#### Mood

IAc is consistently related to anxiety and depression, factors that also influence eating behaviour. Therefore to exclude the possibility that IAc is related to eating behaviour due to covariance with mood, participants were asked to describe their general predisposition using visual analogue scales (VAS) with pairs of adjectives at the ends of 100 mm lines; Composed/Anxious and Elated/Depressed [30]. In addition, it is possible that individuals who are more confident per se would tend to be more confident about their performance on the HBD task. Given that self-confidence might also relate to eating behaviour, participants were asked to report how Unsure/Confident they tend to be using a 100mm VAS [30].

#### Data preparation and analysis

During preliminary analysis a one sample *t* test was used to determine whether performance on the IAc task differed significantly from chance. A median (0.60) split of IAc was made and between subjects *t* tests used to examine whether interoceptive awareness, raw confidence ratings, or interoceptive sensibility differed according to interoceptive ability. In addition a bivariate correlation analysis (Pearsons *r*) confirmed the disassociation between each of the interoception indices.

Pearsons *r* analysis was used to consider the zero order correlation between interoception, eating behaviour and BMI. Cohen’s *d* was used as an effect size measure: d = 0.20 (small effect); d = 0.50 (medium effect) and d = 0.80 (large effect). To test the hypothesis that IAc, IAw, IS and IPE make independent contributions to eating behaviour a series of multiple regressions were conducted. Forkmann et al [31] argue that the objective physiological state (i.e., resting heart rate) constitutes the most basic level of interoceptive processing, as such HR was included as an addition variable. All variables were entered simultaneously. As anxiety, self-confidence and depression have been associated with interoception [21] and emotional eating [32], a final analysis established whether the interoception–eating behaviour associations remained significant after controlling for ratings of anxiety, depression and confidence. Regressions were carried out in a hierarchical fashion where the mood measures were entered in a second step. Cooks distance, with a threshold of N/4, was used to detect possible outliers [33]. This resulted in one case, which had a score of 0.13, being removed. All analysis was carried out using SPSS version 21.

### Results

#### Dissociation between the interoceptive indices

Initially the empirical dissociation between the interoceptive indices was determined. A one sample *t* test confirmed that overall performance on the interoceptive accuracy task was above chance level (Mean = 0.63, SD = 0.19), *t* = 19.890, p<0.0001). Although those with the highest IAc had more confidence in their interoceptive abilities (*t* = 2.974, p<0.005), IAw did not depend on IAc (*t* = 1.249, p = 0.129). Correlation analysis confirmed the dissociation between IAc and IAw (*r* = -0.183, *p* = 0.285). In addition, IS was also not related to IAc (*r* = 0.220, *p* = 0.196) or IAw (*r* = -0.210, *p* = 0.218). However, IPE did correlate with IAw (*r* = -0.389, *p* < 0.01). These findings confirm previous reports of the dissociation between top down and bottom up interoceptive indices [21, 27] and support the view that interoception should not be considered a unitary construct (Table 2).

**Table 2:** Zero order correlations (Pearson’s r) between interoceptive indices.

	IAc	IAw	IS	IPE
**IAw**	-0.183			
**IS**	0.220	-0.210		
**IPE**	-0.093	-0.389*	0.167	
**HR**	-0.377*	0.036	-0.163	-0.027

N = 36. IAc–Interoceptive accuracy, IAw—Interoceptive awareness, IS–Interoceptive sensibility, IPE–Interoceptive prediction error.

* p<0.01. Interoceptive accuracy was not related to interoceptive awareness, sensibility or prediction error. Interoceptive prediction error was associated with interoceptive awareness. Findings support the disassociation between top down and bottom up interoceptive dimensions.

#### Associations between the interoceptive indices, eating behaviour and BMI

**Fig 1:**
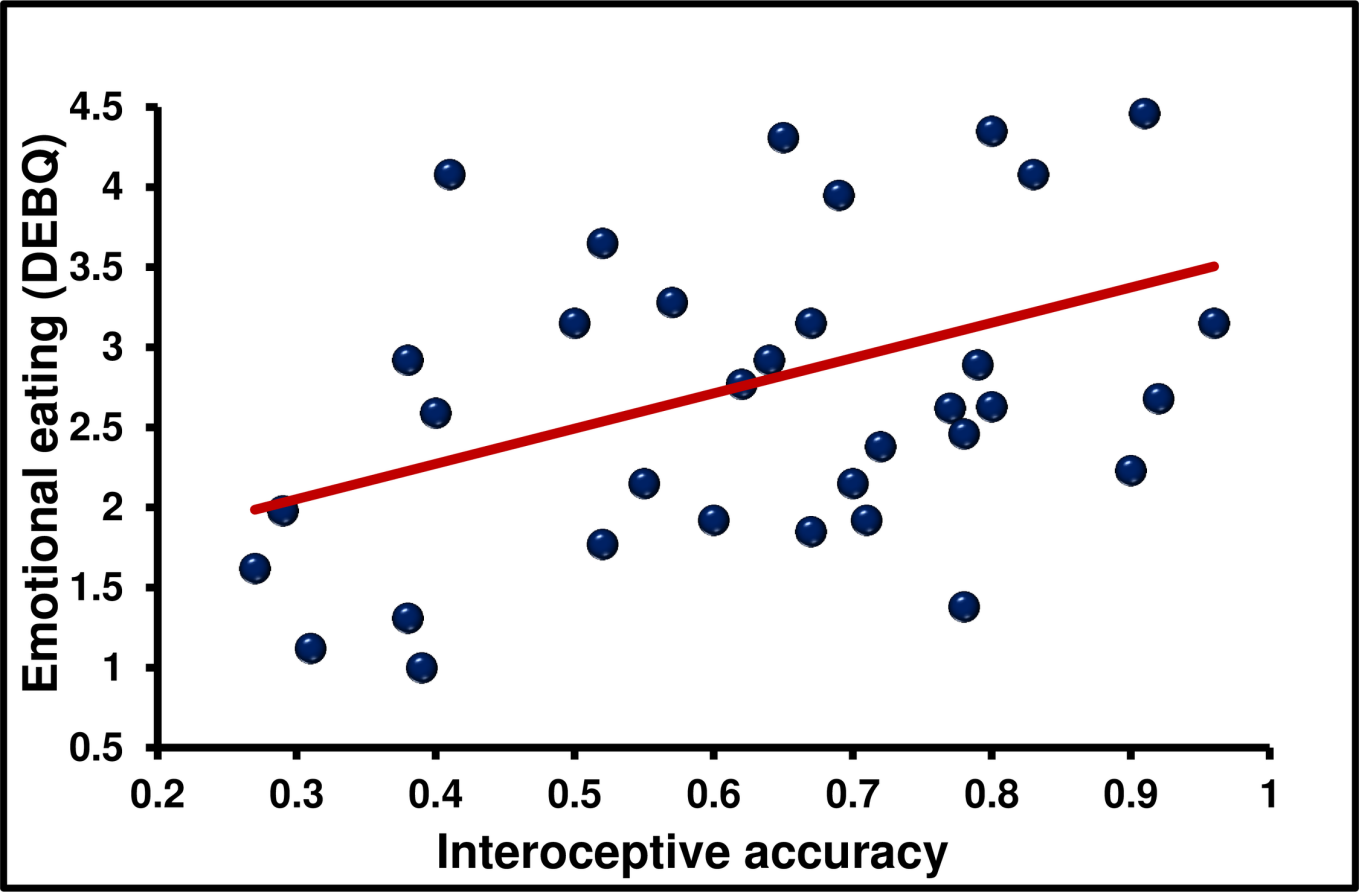
The association between interoceptive accuracy and emotional eating. **N = 36.** Emotional eaters were characterised by higher interoceptive accuracy. but a lower metacognitive insight into their own interoceptive abilities (B).

**Fig 2:**
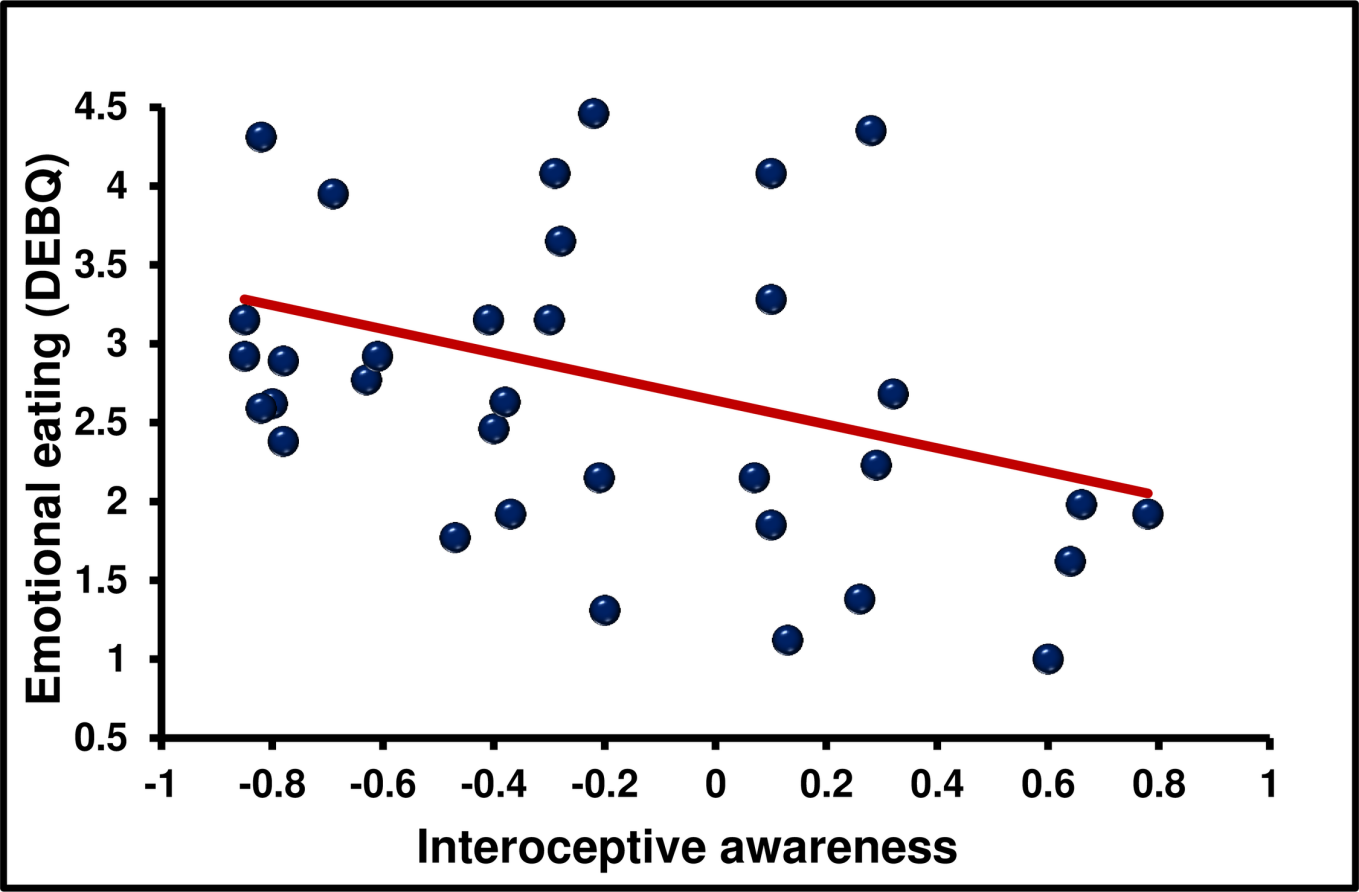
The association between interoceptive awareness and emotional eating. **N = 36.** Emotional eaters were characterised by a lower metacognitive insight into their own interoceptive abilities.

Having established the independence of each interoceptive index their association with eating behaviour and being overweight was considered. There was a positive linear association between IAc and EE (*r* = 0.400, *p* < 0.016, *d* = 0.8) such that those high in EE had higher IAc. Despite having greater interoceptive accuracy, emotional eaters were characterised by lower IAw (*r* = -0.345, *p* < 0.039, *d* = 0.7; Fig 1 and Fig 2); that is they had reduced meta-cognitive insight into their own interoceptive ability. There was a linear association between IPE and EE (*r* = 0.350, *p* < 0.03, *d* = 0.7), such that those with a more accurate belief driven interpretation of their interoceptive information reported less emotional eating.

In line with previous literature [34] EE and EX were positively related (*r* = 0.354, *p* < 0.034, *d* = 0.7) but the association between EX and IAc did not reach significance (*r* = -0.315, *p* < 0.061). External eaters were, however, characterised by lower self-evaluated IS (*r* = -0.431, *p* < 0.006, *d* = 0.9), although neither IPE nor IAw associated with EX (IPE *r* = -0.035, *p* = 0.841, IAw *r* = 0.121, *p* = 0.484).

Restrained eating (the act of abstaining from or avoiding certain foods) was not associated with IAc (*r* = -0.007, *p* = 0.967) nor IS (*r* = -0.097, *p* = 0.573), however, restrained eaters had lower IAw (*r* = -0.395, *p* < 0.039, *d* = 0.7). There were no associations between restrained eating and IPE (*r* = 0.140, *p* = 0.415) (Table 3). Finally the link between interoception and body mass index (BMI) was considered, although, we did not observe any significant associations between BMI and IAc (*r* = 0.003, *p* = 0.988) or IAw *r* = -0.011, *p* = 0.949), IS was positively associated with BMI (*r* = 0.355, *p* < 0.033, *d* = 0.7). IPE did not predict BMI (*r* = -0.086, *p* = 0.616).

**Table 3:** Zero order correlations (Pearson’s r) between interoception and eating behaviour indices.

	IAc	IAw	IS	IPE	HR
**EE**	0.400**	-0.345*	-0.174	0.350*	-0.513**
**EX**	-0.315	0.121	-0.431**	0.035	-0.111
**RE**	-0.007	-0.349*	0.097	0.140	0.001
**BMI**	0.003	-0.011	0.355*	-0.086	-0.229

N = 36. IAc–Interoceptive accuracy, IAw—Interoceptive awareness, IS–Interoceptive sensibility, IPE–Interoceptive prediction error, IPE^2^ –Interoceptive prediction error squared, EE–Emotional eating, EX–External eating, RE–Restrained eating, BMI–Body mass index.

* p<0.05.

** p<0.01. Those high in emotional eating has higher interoceptive accuracy but lower interoceptive awareness. Emotional eating was also associated with having a higher interoceptive prediction error. External eating was associated with having a lower self-evaluated interoceptive sensibility but there were no associations with the objective interoception indices. There was also an association between restrained eating and interoceptive awareness; restrained eaters had lower metacognitive awareness. Body mass index was not associated with the objective interoceptive indices but those with a higher BMI did self–report having higher interoceptive sensibility.

#### The contribution of individual differences in mood

As previously anxiety, self-confidence and depression have been associated with interoception [21], as well as emotional eating [32], we considered whether the interoception–eating behaviour associations might be explained by individual differences in anxiety, depression and self-confidence. Initially the independent associations between interoception, HR and each mood measure were analysed by multiple regression analysis.

Together the interoceptive indices (adjusted R^2^  =  .25, F (5,35)  =  3.403, p < 0.015) accounted for 25% of the variance in anxiety levels. Those with higher IAc were significantly more anxious (β = 0.348, 95% CI LL 2.007, UL 81.905), as were those who self-reported higher levels of interoceptive sensibility (β = 0.330, 95% CI LL 0.007, UL 0.821). Neither IAw (β = -0.066, 95% CI LL -19.443, UL 13.151) nor IPE predicted anxiety (β = -0.213, 95% CI LL -3.446, UL 15.159). In addition, HR was not associated with anxiety levels (β = -0.035, 95% CI LL -0.188, UL 0.151).

In relation to ratings of depression, 36% of the variance was explained by the interoceptive indices and the model reached significance (adjusted R^2^  =  .36, F (5,35)  =  4.826, p < 0.002). Those who rated themselves as more depressed had lower IAc (β = -0.373, 95% CI LL –64.110, UL -5.024) and IS (β = -0.312, 95% CI LL –0.598, UL -0.005). The associations between depression and IAw (β = 0.286, 95% CI LL –1.184, UL 21.484) and depression and IPE (β = 0.088, 95% CI LL -9.341, UL 5.183) were not significant. HR did not predict ratings of depression (β = -0.040, 95% CI LL -0.113, UL 0.102).

We did not observe any significant associations between interoception and self–confidence (adjusted R^2^  =  .008, F (5,35)  =  1.057, p < 0.403) (IAc: β = -0.202, 95% CI LL -60.103, UL 18.542; IAw: β = -0.199, 95% CI LL -24.152, UL 7.931; IPE: β = -0.319, 95% CI LL -1.658, UL 16.655; IS: β = 0.100, 95% CI LL -0.293, UL 0.508; HR: β = -0.008, 95% CI LL -0.171, UL 0.163).

Finally, the unique predictive utility of each interoceptive measure in explaining eating behaviour and BMI, over and above differences in mood, was established. Together the interoceptive indices accounted for 47% of the variance in EE (adjusted R^2^  =  .47, F (5,35)  =  7.493, p < 0.0001). Both IAw (β = -0.321, 95% CI LL –1.220, UL -0.158) and IPE (β = 0.291, 95% CI LL –0.677, UL -0.050) contributed significantly and uniquely to the model. Higher interoceptive awareness but lower prediction error was associated with a lower incidence of EE. The effects of IAc (β = 0.083, 95% CI LL –0.925, UL 1.823) and IS (β = -0.228, 95% CI LL –0.027, UL 0.001) did not make independent contributions to the model, after accounting for the variance associated with IAw and IPE, although HR did (β = -0.599, 95% CI LL –0.020, UL -0.008).

Mood was not related to emotional eating (Anxiety: β = -0.218, 95% CI LL -0.023, UL 0.003; depression: β = 0.010, 95% CI LL –0.017, UL 0.019; confidence: β = 0.008, 95% CI LL -.012, UL 0.013). The effects of IAw and IPE on EE remained significant even after controlling for these aspects of mood, suggesting that the observed effects were not simply a reflection of differences in affect.

A significant 23% of EX was explained by interoception (adjusted R^2^  =  .23, F (5,35)  =  3.135, p < 0.022). IAc made an independent contribution to the model (β = -0.427, 95% CI LL –3.167, UL -0.284); those with a lower IAc reported eating more in response to external cues. The effect of IAw (β = -0.072, 95% CI LL –0.673, UL 0.440) was not significant, and IPE did not predict EX (β = -0.019, 95% CI LL –0.331, UL 0.346). However, those who reported lower IS had a greater propensity towards external eating (β = -0.367, 95% CI LL –0.030, UL -0.001). HR also predicted external eating (β = -0.374, 95% CI LL –0.013, UL -0.001). In the second step anxiety tended to be negatively associated with external eating (β = -0.349 95% CI LL -0.025, UL 0.001), although the effect did not reach significance. Neither depression (β = 0.291, 95% CI LL –0.005, UL 0.030) nor confidence (β = 0.066, 95% CI LL –0.010, UL 0.015) made significant contributions to the model. However, the addition of anxiety, depression, and confidence to the model diminished the association between EX and IS and EX and IAc (IAc: β = -0.218, 95% CI LL –2.419, UL 0.660; IS: β = -0.139, 95% CI LL –0.022, UL 0.010) suggesting these were important cofactors. We did not observe any significant associations between interoception and restrained eating or BMI (S1 File).

## Study 2

### Methods

#### Participants

Thirty seven females between 18 and 28 years of age participated in this study (Table 4). The sample size was based on study one and the same exclusion criteria and pre study instructions were used. BMI ranged from 16.3 to 41.0 (average 22.5) kg/m^2^; 16% of the sample had a BMI < 18.5 and were considered underweight, 67% had a normal BMI between 18.5 and 24.9, 11% of the sample were overweight with a BMI between 25 and 30 and the remaining 6% of the sample were obese with a BMI > 30.

**Table 4:** Descriptive characteristics of the sample for study 2.

Characteristic	Mean (SD)
**N**	37 Females
**Age (years)**	20.56 (1.80)
**Body mass index (kg/m^2^)**	22.56 (4.33)
**Anxiety (VAS)**	31.58 (25.54)
**Depression (VAS)**	30.59 (16.70)
**Confidence (VAS)**	70.27 (21.28)
**Heart rate (BPM)**	76.79 (16.44)
**Emotional eating (EE—DEBQ)**	2.68 (0.74)
**External eating (EX—DEBQ)**	3.04 (0.56)
**Restrained eating (RE—DEBQ)**	2.59 (0.77)

VAS–Visual analogue scale, BPM–Beats per minute, DEBQ–Dutch eating behaviour questionnaire, HBD–Heart beat detection.

#### Procedure

After providing their written informed consent, participants rated their current mood, and conventional Ag/AgCl electrodes and transducers were applied to the subjects and connected to a BIOPAC MP150 and ECG100C amplifier module (BIOPAC, USA). Participants then completed the interoception task as outlined below. Interbeat interval data were monitored throughout the interoception with a sampling rate of 2000Hz. The participants completed the Dutch Eating Behaviour Questionnaire [26] and their height and weight were measured. The procedure was approved by Swansea University department of Psychology ethics committee and was conducted in accordance with the principles laid down by the declaration of Helsinki 2013. All participants provided written informed consent. Eating behaviour, BMI and mood were measured as in study 1.

#### Interoceptive accuracy

Recently it has been argued that heartbeat tracking methods have a false-positive bias in the classification of heartbeat detectors, as participants could use knowledge of their heartbeats to increase their performance. Therefore study used a novel heartbeat discrimination task based on the method of constant stimuli [22]. The task consisted of six R-wave to stimulus intervals [R + 0, R + 100ms, R + 200ms, R + 300ms, R + 400ms and R + 100ms, 110ms, 125ms, 150ms, 200ms, 210 ms, 225ms or 250ms, shuffled randomly until all possibilities were used (i.e. on this trial stimuli were asynchronous with the heartbeat)] and participants viewed eight trials of each R-wave to stimulus interval. Each trial consisted of a circle being presented on the screen for 60ms, and each trial consisted of eight circle presentations triggered by the participant's heartbeat. At the end of each trial, the participants responded by stating whether the series of tones were either *synchronous* or *asynchronous* with her/his heartbeats. Using this paradigm the participants could not use knowledge about their heart rate to guide responses.

#### Data preparation and analysis

χ^2^ was used to determine the distribution of each participants’ responses (synchronous or asynchronous) across trials (R-wave to stimulus intervals). When this χ^2^ test was significant participants qualified as heartbeat detectors. That is, participants were deemed capable of detecting their heartbeat when they preferentially responded ‘synchronous’ to one particular R-wave to stimulus interval over the others [22]. Based on this classification 32.4% of the present sample were considered accurate heartbeat detectors. ANOVA was used to determine the association between anxiety, depression and confidence and interoceptive discrimination ability. In three analysis heartbeat discrimination was the independent variable and mood (anxiety, depression or confidence) the dependent variable. ANCOVA was used to test the hypothesis that accurate heartbeat detectors differ in their eating behaviour. Heartbeat discrimination was entered as the independent variable and eating behaviour the dependent variable. Anxiety, depression and confidence were covariates. All analysis was carried out using SPSS version 21.

### Results

#### Associations between interoceptive discrimination and mood

Individual differences in ability to accurately discriminate ones heartbeat did not predict anxiety (F (1, 35) = 0.267, p = 0.609, ηp2 = 0.008), depression (F (1, 35) = 0.724, p = 0.724, ηp^2^ = 0.004) or confidence (F (1, 35) = 1.494, p = 0.230, ηp^2^ = 0.041) ratings.

#### Associations between interoceptive discrimination, eating behaviour and BMI

**Fig 3:**
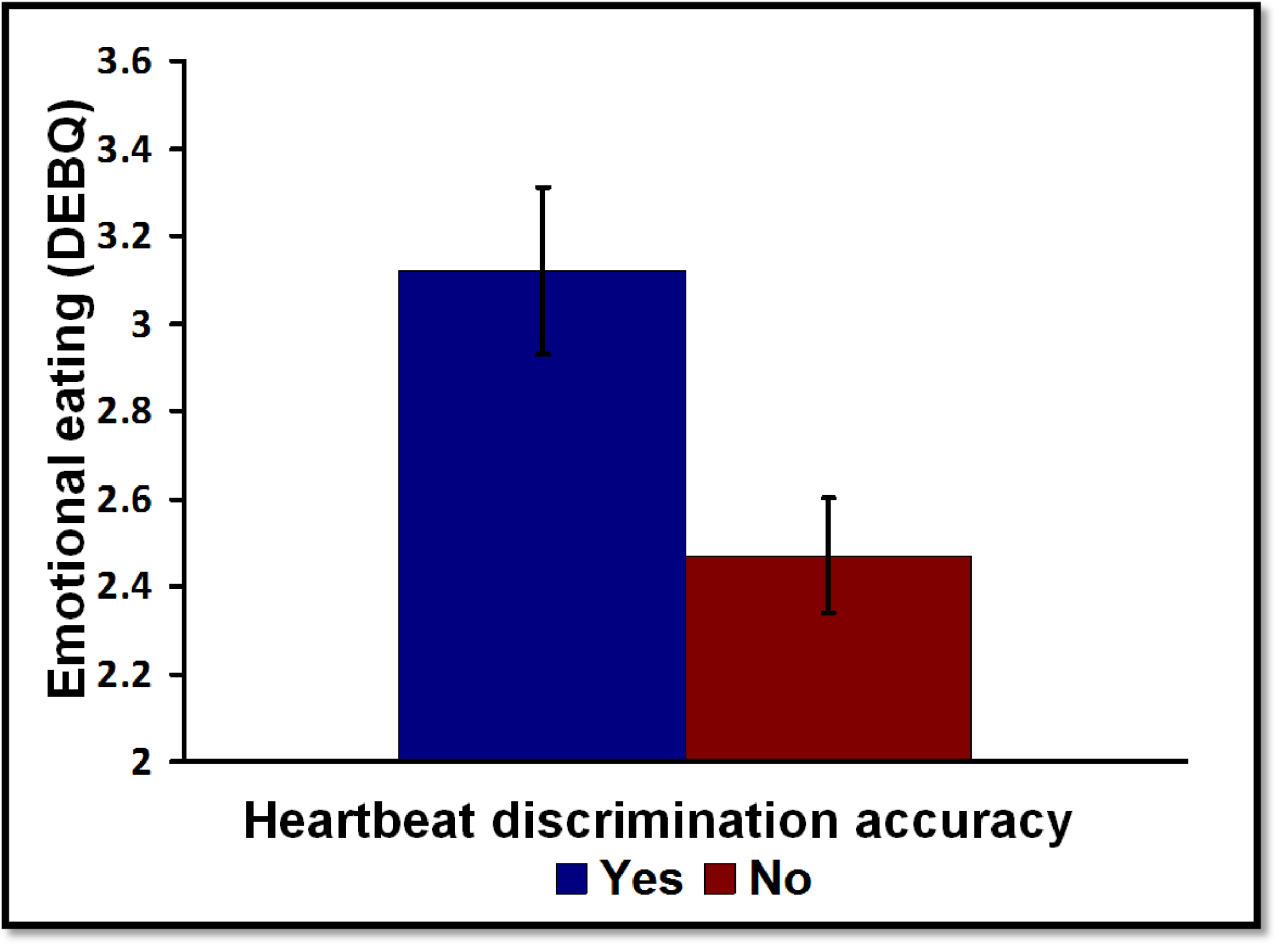
The association between heartbeat discrimination and emotional eating. **N = 37.** Those where were able to accurately discriminate their heartbeat had a greater propensity for emotional eating.

Those who were able to accurately discriminate their heartbeat reported a significantly greater propensity towards emotional eating (F (1, 35) = 7.369, p < 0.01, ηp^2^ = 0.174; Fig 3). Conversely an ability to accurately discriminate one’s heartbeat was associated lower external eating, although this effect was smaller (F (1, 35) = 3.832, p < 0.05, ηp^2^ = 0.099: Detector mean (SE) 3.16(0.10), Non-detector mean (SE) 2.79 (0.15)). No associations were observed for restrained eating (F (1, 35) = 0.703, p = 0.401, ηp^2^ = 0.020) or BMI (F (1, 35) = 0.503, p = 0.483, ηp^2^ = 0.014). Those who were most depressed rated themselves as more likely to eat for external reasons (F (1, 35) = 4.077, p < 0.051, ηp^2^ = 0.113) but mood was not related to any other aspect of eating behaviour and did not influence the association with heartbeat detection.

## Discussion

The main objective was to establish which facets of interoception are related to eating behaviour and being overweight. Key findings were that; (1) in the present sample IAc, IAw, IS and IPE were dissociable dimensions of interoception (Table 2); (2) EE is characterised by increased IAc but reduced IAw and IPE (Table 3 and Fig 1 and Fig 2); (3) an EX eating style was associated with lower IAc and IS; (4) the link between EE and interoception could not be accounted for by individual differences in mood. (5) The association between IAc and EE and IAc and EX were confirmed in study two using a novel heart beat discrimination task (Fig 3).

The present findings support the validity of the interoceptive framework put forward by Garfinkel et al. [27]; that is IAc and IAw are distinguishable processes underlying interoceptive ability. Furthermore we provide empirical support that belief driven interpretation of ones interoceptive information is dissociable from the metacognitive IAw component. It is important to note that whereas Garfinkel et al. [27] used the body perception questionnaire (BPQ) to assess interoceptive sensibility, the current study employed the MAIA. Thus, although IS was operationalized with different self-report instruments, similar results were obtained, confirming the assumption that IS is distinct from IAc, irrespective of the self-report method used.

The finding that IAw represents a distinct interoceptive facet is important as the previous literature associating interoception and eating behaviour has exclusively focused on either IS or IAc. Therefore, this is the first study to establish a role for IAw in EE.

Metacognitive processes, such as theory of mind [35], mentalization [36], alexithymia [37] and self—regulation [38] have all been related to symptom severity in those with disordered eating. However, a coherent understanding of the mechanisms underlying these deficits has remained elusive. It is increasingly recognised that human cognition is essentially embodied; that is visceral afferent signals are an essential component of one’s current cognitive and emotional content. Therefore, individual differences in the mental representation of interoceptive signals (IAw) are likely to play an important role in self-regulated behaviour.

Indeed, self-regulation first requires that you actively monitor and are aware of on-going processes. Those low in interoceptive awareness might find it difficult to actively monitor and therefore take appropriate action, to adjust their interoceptive signals. Although speculative, it is plausible to suggest that deficits in interoceptive awareness underlie the poor self-regulatory abilities commonly observed in EE [38]. An implication of this finding is that patients with eating disorders could misinterpret or confound emotional and visceral / satiety signals. Future research should explore this possibility in clinical populations.

From the perspective of interoceptive inference [6] emotions arise as a result of inferences about the cause of on-going interoceptive signals. Thus it is plausible that those low in interoceptive awareness might misinfer the source of their interoceptive signals, hindering their ability to effectively self-regulate. This is in line with a literature which finds an association between alexithymia and disordered eating [37], including emotional eating [39].

It is noteworthy that the neural basis of metacognition shares considerable overlap with eating behaviour, in particular the lateral and medial pre frontal cortices (PFC) are consistently implicated in both [40, 41]. In relation to interoception (IAc), similar overlaps occur: for example the medial PFC, anterior and posterior cingulate and anterior insula cortices are linked to both interoception [42, 43] and EE [44, 45]. In addition, cardiac sensations have recently been linked with reduced activation in the putamen and ventromedial PFC response to food images in anorexia nervosa. Of note, the agranular visceromotor cortices—including the cingulate cortex, the posterior ventral medial PFC, the posterior orbitofrontal cortex, and the most ventral portions of the anterior insula, have been posited as key brain regions that generate interoceptive predictions and prediction errors [42]. This is in line with a body of neuroimaging studies which find differential anticipatory activations in these brain regions according to BMI [41]. Future research would profit from considering the role of metacognitive IAw when examining the neural basis of eating behaviour.

Despite lower IAw, EE were characterised by higher IAc, in line with a previous study that also reported a positive association between IAc and EE in overweight children [46]. This effect was found in both study one and study two, using the heartbeat tracking and heartbeat detection tasks. There is evidence indicating that individuals with higher interoceptive accuracy experience more emotional arousal despite having similar objective physiological responses. For example, Dunn et al. [47] measured participants’ arousal to emotional pictures both objectively (through heart rate change and skin conductance), and subjectively (by self-report). Those with high interoceptive accuracy reported higher arousal than people with low interoceptive accuracy, despite having identical objective changes in physiological variables. Similarly, strong P300 evoked potentials (a marker of emotional processing), as well as greater self-reported arousal, have been reported in people with high interoceptive accuracy when responding to emotional pictures [48].

In relation to eating behaviour a plausible hypothesis is that emotional eaters are characterised by high interoceptive accuracy and as such sense more strongly physiological changes associated with emotion. In line with this point of view, disordered eating patterns are often associated with high levels of perceived emotion intensity, as well as difficulty regulating affect [49]. For instance, neuroticism (a personality trait characterized by anxiety, fear, moodiness, worry, envy, frustration, jealousy and loneliness) is reliably associated with EE [41]. Interestingly Terasawa et al. [50] reported recently that activity within the right anterior insula cortex (a brain region thought to play a role in interoception) positively correlated with neuroticism. Taken together these findings suggest that aberrant interoceptive signalling may increase emotional sensitivity in EE and contribute to their inability to emotionally self-regulate.

However, in contrast to the present findings, Herbert et al. [8] used the intuitive eating scale (IES) [51] to assess the link between IAc and eating behaviour. They found that those who reported “eating for physical rather than emotional reasons” had higher IAc. An important consideration is that ‘eating for physical’ and ‘eating for emotional’ reasons are not necessarily diametric behaviours as implied by the IES. Indeed IAc, measured according to heartbeat perception tasks, might be differentially related to these eating styles. On the other hand, the emotional eating scale of the DEBQ provides a relatively pure measure of EE, although when an aggregate score is taken it does not differentiate between emotions. These considerations will be important avenues for future research.

It is also worth noting that the vast majority research linking interoception and eating behaviour has been conducted in clinical populations resulting in inconsistent findings. For example, those with anorexia have been shown to have reduced heartbeat perception [11], yet there is higher gastric satiation [52] and a heightened neural response during an aversive breathing load [53]. In those with bulimia, barostat studies show significantly decreased stomach sensitivity to fullness [54], two out of three studies report no heartbeat perception deficits [54], while neural activity is increased in anticipation of an aversive breathing load [55]. As clinical eating disorders are heterogeneous, our aim was to study specific eating behaviour traits in an attempt to shed light on these inconsistencies.

Importantly, the degree of EE may vary across eating disorder sub-types and individuals. For example, while those with bulimia report a higher propensity for EE than general dieters, those with anorexia report considerably less [56], potentially explaining the aforementioned inconsistencies. In addition when sampled ecologically, it is emotion rather than hunger per se that appears to precipitate a binge eating episode [57, 58]. In this respect it may be pertinent that heartbeat tracking / detection tasks tap individual differences in interoceptive processing, as it relates to emotional experience rather than hunger and satiety.

Interestingly high IAc was also associated with a lower propensity towards EX. These findings are in line with a body of literature highlighting the importance of exteroceptive signals in contextualising interoceptive processes. For instance, those with low interoceptive accuracy experience a stronger rubber hand illusion [59], suggesting that they are more sensitive to exteroceptive information. In support of this Mata et al. [60] reported that in obese adolescents, insula activity during a risk taking task was positively associated with interoceptive accuracy but negatively associated with external eating. Taken together these findings support the view that by virtue of their reduced interoceptive signalling, EX may rely more on exteroceptive food cues when making food related decisions.

Finally, an interesting observation is that EE was also associated with IPE (Table 3). Garfinkel et al. [21] recently reported that those with autism (a disorder characterised by an inability to process emotions of self or other) tended to overestimate their interoceptive ability; an effect associated with a shallower emotional sensitivity but greater anxiety. These mismatches between subjective and objective performance (IPE) were interpreted within the interoceptive inference framework as a failure to properly incorporate ‘bottom-up’ interoceptive signals, when updating ‘top-down’ interoceptive predictions that inform subjective judgments of sensibility. This is an interesting proposition in relation to eating behaviour as historically researchers in the field have tended to focus their attention exclusively on either ‘top down cognitive control’ or ‘bottom up gut-brain signalling’. The present findings suggest that attention might be better directed towards understanding how these processes coalesce to influence to eating behaviour.

The limitations of the present study should be considered. Currently it is unclear the extent to which interoception is modality specific and more specific interoception measures focusing on the gastric tract are required. Recently, Herbert et al. [61] found that heart beat perception accuracy was inversely correlated with ingested water volume and gastric activity, but not subjective ratings of fullness, nausea and mood. In addition, the amount of water consumed during a water load test was positively related to the bulimia subscale on the EDI [62]. These findings suggest that there is some degree of overlap in the sensitivity of interoceptive processes across modalities, although more research is required to confirm this contention. In addition, although the present study addressed important questions regarding the inconsistencies in the literature connecting interoception and eating behaviour (i.e. comorbid anxiety / depression and the role of interoceptive inference) methodological differences (e.g. the use of different heartbeat detection tasks or eating disorder-specific vs. non-specific measures) might also explain these inconsistencies. Furthermore the present findings are based on VAS measurements of mood. Although such measures have been validated against a range of psychometric tests [63] future research might utilise the BDI-II, STAI-S. or PANAS to confirm the present results. Given the increased recognition of male eating disorders further research might also consider whether such effects are also present in male samples.

## Conclusion

The present results indicated that a complex pattern of interoceptive processing underlies aberrant eating behaviour. EE had a higher IAc but lower metacognitive IAw which may impede their attempts at self–regulation. On the other hand EX has lower IAc, supporting the view that EX may be more sensitive to exteroceptive food cues. EE were also defined by an interoceptive prediction error which may be interpreted as an inability to assimilate ‘bottom up’ and update ‘top down’ interoceptive predictions. It is now evident that interoception can no longer be viewed as a largely ‘bottom up’ gathering of evidence but rather should be viewed as a top-down anticipatory neural representation that predicts the causes of sensory signals. As such, understanding the associations between interoception, eating behaviour and obesity will require a multifaceted approach. Further research aimed at understanding the inferential nature of interoceptive processing will likely shed light on mechanisms underlying pathological eating behaviour and pave the way towards innovative treatment methods.

## Supporting information

S1 File Contains correlations between each MAIA and DEBQ subscale and the analysis concerning restrained eating, BMI and interoception. (DOCX)
