# Supplementary material for: Getting to the heart of the matter: Does aberrant interoceptive processing contribute towards emotional eating?
Source: PLoS One. 2017 Oct 18;12(10):e0186312. doi: 10.1371/journal.pone.0186312 (PMC5646794; doi:10.1371/journal.pone.0186312)
Supplement: S1 File — (DOCX) [file pone.0186312.s001.docx]

**SUPPLEMENTARY DATA**

**Getting to the heart of the matter: Does aberrant interoceptive processing contribute towards emotional eating?**

**Young et al. 2017**

**S1 Table 1. Correlations between the individual subscales of the Multidimensional Assessment of Interoceptive Awareness Questionnaire, eating behaviour and BMI.**

|  | **Noticing** | **Not distracting** | **Not worrying** | **Attention regulation** | **Emotional awareness** | **Self – regulation** | **Body listening** | **Trust** |
| --- | --- | --- | --- | --- | --- | --- | --- | --- |
| **EE** | -.051 | .108 | **-.545**** | .216 | -.010 | .120 | **-.340*** | -.187 |
| **EX** | **-.483*** | .066 | **-.653**** | -.231 | -.180 | -.195 | **-.324*** | -.281 |
| **RE** | -.117 | .304 | -.182 | .024 | .043 | -.221 | -.038 | **-.348*** |
| **BMI** | .268 | -.116 | -.061 | .310 | **.462*** | -.043 | .077 | -.015 |

N= 36. EE – Emotional eating, EX – External eating, RE – Restrained eating, BMI – Body mass index,. * p<0.05. ** p<0.01.

Emotional and external eaters were characterised by a tendency to experience distress in response to one’s physiological sensations, and a lack of an ability to listen to the body for insight. External eating was also linked to a lack of awareness to physiological signals per se. Restrained eaters tended to lack trust in their bodily signals while those with the highest BMI reported having a greater physiological sensations during emotional experience.

**Associations between restrained eating and interoception after controlling for mood**

The model was not significant (adjusted R^2^ = .01, F (5,35) = 0.887, p = 0.502) and we did not observe any significant associations between restrained eating and interoception: IAc (β = -.169, 95% CI LL –2.814, UL 1.345), IAw (β = -.279, 95% CI LL –1.242, UL 0.278), IPE (β = -.001, 95% CI LL –0.439, UL 0.442) and IS (β = .013, 95% CI LL –0.019, UL 0.020). Mood did not predict restrained eating (anxiety: β = -.111, 95% CI LL –0.021, UL 0.013; depression: β = -.271, 95% CI LL –0.036, UL 0.010; confidence: β = .019, 95% CI LL –0.016, UL 0.018).

**Associations between body mass index and interoception after controlling for mood**

Similarly, when BMI was considered the model was not significant (adjusted R^2^ = .04, F (5,35) = 1.324, p = 0.281). Neither IAc (β = -.317, 95% CI LL –16.715, UL 2.562), IAw (β = .126, 95% CI LL –2.406, UL 4.639), or IPE (β = .154, 95% CI LL –2.830, UL 1.255) predicted BMI. Anxiety predicted BMI (β = .451, 95% CI LL .006, UL 0161) but depression (β = -.066, 95% CI LL –0.124, UL 0.092) and confidence (β = 0.137, 95% CI LL –0.048, UL 0.107) did not.
